# Correlation of plasma concentration and adverse effects of bosutinib: standard dose or dose-escalation regimens of bosutinib treatment for patients with chronic myeloid leukemia

**DOI:** 10.1186/s40164-018-0101-1

**Published:** 2018-04-13

**Authors:** Akiko Mita, Maiko Abumiya, Masatomo Miura, Takenori Niioka, Saori Takahashi, Tomoko Yoshioka, Yoshihiro Kameoka, Naoto Takahashi

**Affiliations:** 1 Department of Hematology, Nephrology, and Rheumatology, Akita University Graduate School of Medicine, 1-1-1 Hondo, Akita City, Akita 010-8543 Japan; 2 Department of Pharmacy, Akita University Hospital, Akita, Japan; 3 Clinical Research Promotion and Support Center, Akita University Hospital, Akita, Japan

**Keywords:** Bosutinib, Diarrhea, Liver dysfunction, Trough concentration, Chronic myeloid leukemia

## Abstract

**Purpose:**

To investigate the exposure-toxicity relationship of bosutinib and to identify the target trough plasma concentration (C_0_).

**Methods:**

The toxicity and C_0_ of bosutinib in Japanese chronic myeloid leukemia (CML) patients were monitored every 2 weeks for the first 3 months of treatment, and subsequently once a month during the 6 months after beginning 500 mg/day of standard dose (SD group, n = 10) or beginning 100 mg/day and increased by 100 mg every 2 weeks of dose escalation (DE group, n = 15).

**Results:**

Nine of 10 patients (90%) in the SD group were not able to continue bosutinib therapy without interruption due to adverse events, compared to 2 patients (13.5%) in the DE group. The total duration of treatment interruption was 35 and 14 days in the SD and DE groups, respectively. The median time until liver dysfunction or diarrhea was day 28 and day 1 in the SD group, and day 53.5 and day 19 in the DE group, respectively. The cumulative dose of bosutinib was comparable between the SD and DE groups (51,700 vs. 53,550 mg, respectively). At 6 months, the median C_0_ was 63.7 ng/mL and 63.0 ng/mL in the SD and DE groups, respectively. Liver dysfunction (all grades) and diarrhea (> grade 2) were prevalent in quartile 4 of C_0_ (> 91.0 ng/mL), as calculated by the total C_0_ distribution.

**Conclusions:**

The DE regimen was better suited to avoid treatment interruption. The daily dose of bosutinib might be adjusted based on target C_0_ to avoid adverse events by therapeutic drug monitoring in general practice.

## Background

Bosutinib is a second-generation tyrosine kinase inhibitor (TKI) for the Src and Abelson (Abl) kinases, which was originally developed for the treatment of patients with chronic phase (CP), accelerated phase, and blast phase Philadelphia chromosome-positive chronic myeloid leukemia (CML), who developed resistance or intolerance to prior TKIs such as imatinib, nilotinib, and dasatinib, or for whom other TKIs are not regarded as appropriate treatment options due to comorbidities [1–3]. In addition, in the Bosutinib Efficacy and Safety in Newly Diagnosed CML (BELA) trial and the Bosutinib trial in First-line chrOnic myelogenous leukemia tREatment (BFORE) trial, the achievement rate of a major molecular response (MMR) was significantly higher, and the median times to achieve MMR was significantly shorter, in patients taking bosutinib than in those taking imatinib [4, 5]. Recently, bosutinib was approved as a first-line therapy for CML patients by the US Food and Drug Administration (FDA).

In TKI therapy for CML patients, therapeutic drug monitoring (TDM) is a new strategy for optimizing dosage to achieve faster and deeper responses, such as an MMR [6]. Although this strategy could increase efficacy and/or decrease toxicity from TKI therapy, it is important to confirm the proposed target plasma concentrations, especially in medications with narrow therapeutic windows. Namely, the dosage of TKI could be adjusted based on target plasma concentration to maximize efficacy and to minimize the incidence of adverse events (AEs). For CML patients taking imatinib, the target trough plasma concentration (C_0_) are reported to be set above 1000 ng/mL to maximize efficacy. Bosutinib exposure and C_0_ calculated from pooled plasma samples within 1 month after initiation of bosutinib therapy have also been reported to be associated with an achievement rate of MMR within 12 months [7]; however, target bosutinib C_0_ is uncertain. In addition, patients with a higher bosutinib C_0_ tended to have an elevated risk of bosutinib-induced diarrhea [8], which is the most frequent adverse event due to bosutinib [1–5, 7–10]. These results show that TDM may be necessary for bosutinib. However, these pharmacokinetic results from phase I–III trials [7, 8] were limited in that they evaluated clinical responses, such as MMR at 12 months, as well as toxicities, by only examining bosutinib exposure within 1 month after administration. The dose reduction of bosutinib is typically a result of AEs, which was shown to occur in about 39% of patients assessed for at least 1 year during a phase III trial [4].

In the present study, we monitored C_0_ of bosutinib in Japanese CML patients during the 6 months after beginning the standard dose or dose escalation of bosutinib, and investigated the relationships between bosutinib exposure and toxicities, especially diarrhea and liver dysfunction.

## Methods

### Patients and protocols

The 25 Japanese CML patients enrolled in this study were either resistant to, or intolerant of, imatinib, except for 1 patient with newly diagnosed de novo CML. All patients were treated at the Akita University Hospital between July 2009 and August 2017. The study was conducted according to the principles of the Declaration of Helsinki. This study was approved by the Ethics Committee of Akita University Graduate School of Medicine (no. 1140). Each participant provided informed consent and signed a human subject institutional review board consent form. The first 10 patients among the 25 patients were treated with the standard dose of bosutinib (500 mg, once daily) and the other 15 patients were treated using a dose escalation regimen. Patient characteristics at the initial bosutinib administration (baseline) are listed in Table 1. During the study period, patients were not administered proton pump inhibitors and/or drugs or foods known to affect CYP3A or P-glycoprotein function. Details of the standard dose (SD) regimen included the following. Initially, bosutinib was orally administered 500 mg once daily at 08:00 after breakfast. After that, the maintenance dosage of bosutinib was adjusted in 100-mg increments, based on patient condition. Basically, dose reductions of bosutinib were conducted based on AE grade, for each side effect. For example, at the time of onset for grade 1–2 AEs, the bosutinib therapy was continued by using the same dose with an appropriate supporting therapy while observing each patient’s condition. At the time of onset for grade 3 AEs, the bosutinib therapy was temporarily discontinued until each AE was asymptomatic. After that, the patient received the same dose, or the previous dose reduced by 100 mg.

**Table 1:** Clinical characteristics of patients administered bosutinib

Characteristics	Frequency		*P* value
	Standard dose	Dose escalation	
Total number	10	15	
Female: male	5:5	8:7	
Age (year)	63.0 (34.0–78.0)	55.0 (19.0–81.0)	0.183
Phase, CP: AP: BP	7:2:1	15:0:0	
Reason of taking bosutinib, TKI-R: TKI-Int: de novo CML	4:5:1	9:6:0	
Body weight (kg)	59.9 (40.8–69.1)	53.0 (44.1–86.3)	0.889
Laboratory test values			
White blood cell (*10^3^/mm^3^)	8.5 (3.6–190.7)	6.1 (1.3–13.8)	0.332
Hemoglobin (g/dL)	10.3 (8.2–14.2)	12.1 (8.3–14.8)	0.052
Platelet (*10^4^/mm^3^)	29.5 (12.1–326.0)	19.4 (12.8–162.0)	0.120
Aspartate transaminase (IU/L)	23.0 (14.0–56.0)	24.0 (10.0–47.0)	0.487
Alanine transaminase (IU/L)	17.5 (9.0–65.0)	23.0 (7.0–34.0)	0.232
Serum albumin (g/dL)	4.1 (3.7–4.3)	4.2 (3.0–5.2)	0.179
Total bilirubin (mg/dL)	0.6 (0.2–0.9)	0.6 (0.3–2.7)	0.557
Serum creatinine (mg/dL)	0.8 (0.5–0.9)	0.7 (0.4–1.7)	0.803
Lipase (IU/L)	21.5 (5.0–75.0)	18.0 (6.0–38.0)	0.781

Data presented as number or median (minimum–maximum)

*TKI* tyrosine kinase inhibitor, *R* resistance, *Int* intolerance

Details of the dose escalation (DE) regimen included the following. Initially, bosutinib was orally administered at 100 mg once daily at 08:00 after breakfast during the first 2 weeks of therapy initiation. On day 15 after beginning therapy, bosutinib was increased to 200 mg once daily based on each patient’s condition; subsequently, every 2 weeks, bosutinib was increased by 100 mg to a maximum of 500 mg/day based on patient condition. The final bosutinib dose is shown in Table 2. For AEs, toxicity grades were determined using the Common Terminology Criteria for Adverse Events (CTCAE) version 4.0.

**Table 2:** Patient disposition and dose intensity for the study treatment

All patients during day 1 to day 180	Standard dose (n = 10)	Dose escalation (n = 15)	*P* value
Interruption, n (%)	9 (90.0%)	2 (13.3%)	< 0.001
Diarrhea	9 (90.0%)	0 (0.0%)	
Liver dysfunctions	5 (50.0%)	2 (13.3%)	
Skin rash	2 (20.0%)	0 (0.0%)	
Cytopenia	3 (30.0%)	0 (0.0%)	
Elevation of lipase	1 (10.0%)	0 (0.0%)	
Median (range) day to the first interruption of bosutinib	17.5 (8–180)	180 (35–180)	< 0.001
Total median (range) duration of treatment interruption (days)	35 (8–81)	14 (14–14)	0.013
Dose reduction, n (%)	8 (80.0%)	8 (53.3%)	0.182
Discontinuation of treatment, n (%)	3 (30.0%)	1 (6.6%)	0.127
Adverse events	2 (20.0%)^a^	1 (6.6%)	
Progression disease	1 (10.0%)	0 (0.0%)	

On day 180 after beginning therapy	Standard dose (n = 7)	Dose escalation (n = 14)	*P* value
Final dose: 100/200/300/400/500 mg QD, n	0/0/5/1/1 (mean 343 mg/day)	1/2/4/4/3 (mean 346 mg/day)	0.403
Median (range) duration of administration (days)	157.0 (99–180)	180.0 (166–180)	< 0.001
Median (range) cumulative dose for 6 months (mg)	51,700 (32,800–90,000)	53,550 (29,700–75,600)	1.000
Median (range) dose intensity of bosutinib (mg/day)	309.8 (182.2–500.0)	295.0 (165.0–420.0)	1.000
Median (range) plasma trough concentration (ng/mL)	63.7 (31.9–126.0) (mean = 75.1)	63.0 (31.4–113.0) (mean = 65.8)	0.588

^a^Two patients discontinued bosutinib due to pancytopenia (n = 1) and elevation of lipase (n = 1)

During bosutinib administration, patients had blood collected at regular intervals for the monitoring of side effects. Namely, bosutinib C_0_ was monitored at just prior to oral administration or 25 ± 1 h after administration for outpatients, in 2-week intervals for the first 3 months after beginning treatment, then once a month during the rest of the 6-month treatment period. A total of 136 samples of bosutinib plasma concentrations were collected in this study. Plasma was isolated by centrifugation at 1900×*g* for 15 min and was stored at − 40 °C until analyzed.

### Analysis of bosutinib plasma concentrations

Plasma concentrations of bosutinib were measured by high-performance liquid chromatography (HPLC). Following the addition of erlotinib (5 ng/10 µL of methanol) as an internal standard to a 100 µL plasma sample, the plasma sample was diluted with 900 µL of water and vortexed for 30 s. This mixture was then applied to an Oasis HLB extraction cartridge that had been activated previously with methanol and water (1.0 mL each). The cartridge was then washed with 1.0 mL of water and 1.0 mL of 60% methanol in water and eluted with 1.0 mL of 100% methanol. Eluates were dried by vortex-vacuum evaporation at 70 °C using a rotary evaporator (as-1 cve-2as, Osaka, Japan). The resulting residue was then dissolved in 20 µL of methanol and vortexed for 30 s; 20 µL of the mobile phase was added to the sample, and the sample was vortexed for another 30 s. A 20 µL aliquot of the sample was then processed by HPLC. The HPLC system comprised of a PU-2080 plus chromatography pump (Jasco, Tokyo, Japan) equipped with a capcell pak c18 mg П (250 mm × 4.6 mm internal diameter, Shiseido, Tokyo, Japan) HPLC column, a UV-2075 light source, and an ultraviolet detector (Jasco). The mobile phase was 0.5% KH_2_PO_4_ (pH3.5)–acetonitrile–methanol (60:35:5, v/v/v), which was degassed in an ultrasonic bath prior to use. The flow-rate was 0.5 mL/min at ambient temperature and sample detection was conducted at 250 nm.

The calibration line of bosutinib in plasma was linear over the concentration range of 10–500 ng/mL. The extraction recovery value of bosutinib in this concentration range was 86–90%. The coefficient of variations (CVs) and accuracies for the intra- and inter-day assays at concentrations of 10–500 ng/mL of bosutinib was less than 11.3% and within 9.8%, respectively. The limit of quantification of bosutinib was 10 ng/mL.

### Statistical analysis

The Shapiro–Wilk test was used to assess the distribution of all patient data. The clinical characteristics of patients were expressed as the number or median value (range: minimum–maximum). The daily dose and C_0_ of bosutinib were expressed as the median and range. The Chi square test was used to examine the difference in categorical data. The Mann–Whitney U test was used to determine the difference in continuous variables between treatment groups. Spearman’s rank correlation analysis was used to assess correlations with the C_0_, or the dose-adjusted C_0_, of bosutinib with all results being expressed as correlation coefficients (r). Stepwise, multiple linear regression analysis was performed to determine the effect of all factors in the univariate analysis. The rate of side effects after beginning treatment between the two regimens was estimated using the Kaplan–Meier method and compared using the stratified log-rank test. *P* values less than 0.05 were considered statistically significant. Statistical analyses were performed using SPSS version 20.0 for Windows (IBM Corp., Armonk, NY, USA).

## Results

Clinical characteristics at the initial bosutinib administration (baseline) are shown in Table 1. There were no patients with serious renal or hepatic dysfunction. On the SD and DE regimens, the median patient age was 63.0 and 55.0 years, respectively, and the median body weight was 59.9 and 53.0 kg, respectively. No significant difference was observed in any laboratory value at baseline between the two regimens, except for the hemoglobin value, which was associated with CML phase.

In nine of 10 patients (90%) on the SD regimen, the bosutinib treatment was interrupted due to AEs. Subsequently, 8 patients were given a lower daily dose of bosutinib (Table 2). The median time to the first treatment interruption after beginning 500 mg/day of bosutinib was 17.5 days. During 6 months, there was a median total interruption duration of 35 days. Moreover, after beginning the 500 mg/day treatment, although dosage was adjusted based on the AE grade, 2 patients discontinued bosutinib therapy due to grade 3 elevation of lipase levels and grade 3 pancytopenia. In addition, another patient discontinued bosutinib therapy due to disease progression. Among 7 patients who were able to continue the bosutinib treatment, the final daily doses of bosutinib were 300 mg (5 patients), 400 mg (1 patient), and 500 mg (1 patient), and the mean daily dose was of 343 mg/day (median, 300 mg/day). Only 1 patient could be treated during the 6-month period using the standard 500 mg daily dose of bosutinib on a continuous basis. Consequently, the median C_0_ of bosutinib was 63.7 ng/mL (Table 2).

In 2 of 15 patients (13.3%) on the DE regimen, the bosutinib treatment was interrupted due to liver dysfunction; however, after re-administration of bosutinib, 14 of 15 patients could be treated continuously during the treatment period after beginning the 100 mg/day escalated dose. Only 1 patients showed symptoms due to the withdrawal of bosutinib, which included severe liver dysfunction (grade 4) for 14 days. The frequency of interruption was significantly lower in the DE than the SD regimen (*P* < 0.001). For the DE regimen, the final daily doses of bosutinib were 100 mg (1 patient), 200 mg (2 patients), 300 mg (4 patients), 400 mg (4 patients), and 500 mg (3 patients), with a mean dose of 346 mg/day (median, 400 mg/day) (Table 2). The cumulative dose of bosutinib for the entire treatment period was similar between the SD and DE treatment groups (51,700 mg vs. 53,550 mg, respectively). In addition, the median C_0_ was 63.0 ng/mL, which was nearly identical to the 63.7 ng/mL value of the SD group.

**Fig. 1:**
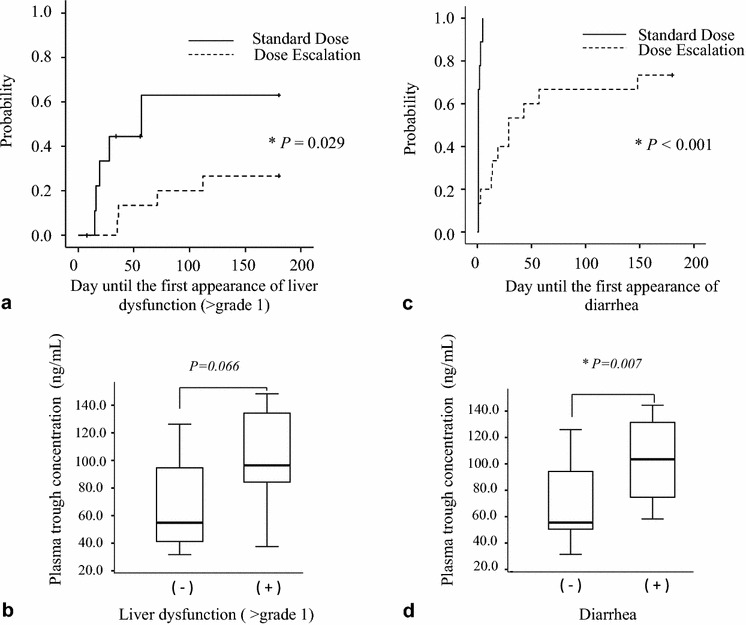
**a** Kaplan–Meier curve for the first onset of liver dysfunction (> grade 1). The median time of the first appearance of liver dysfunction was 57 days in patients receiving the standard dose regimen; however, that was not reached in patients on dose escalation regimen (*P* = 0.029, log-rank test). **b** Comparison of plasma trough concentration of bosutinib in patients with liver dysfunction (> grade 1, n = 9), as well as those without (n = 16). There was no statistically significant difference in the plasma trough concentration between the two groups (*P* = 0.066, Mann–Whitney U test). **c** Kaplan–Meier curve for the first onset of diarrhea. The median time of the first appearance of diarrhea was 1 day in patients receiving the standard dose regimen and 29 days in patients receiving the dose escalation regimen (*P* < 0.001, log-rank test). **d** Comparison of plasma trough concentration of bosutinib in patients with delayed/prolonged diarrhea, which occurred after day 14 (n = 11), as well as those without symptoms (n = 14). There was a statistically significant difference in plasma trough concentration between these two groups (*P* = 0.007, Mann–Whitney U test)

In this study, 5 of 10 patients (50.0%) experienced liver dysfunction (> grade 1) between days 19 and 57 (median, day 28) for the SD regimen, and 4 of 15 patients (26.7%) experienced liver dysfunction between days 35 and 112 (median, day 53.5) in the DE regimen (Table 3). The Kaplan–Meier curve for the first appearance of liver dysfunction (> grade 1) is shown in Fig. 1a. Although the cumulative dose until the onset of liver dysfunction or the dose at the onset of liver dysfunction was not significant different between the SD and DE groups (Table 3), the cumulative incidence of liver dysfunction was significantly higher in the SD group (*P* = 0.029, log-rank test). Differences in plasma C_0_ between patients with liver dysfunction and patients without liver dysfunction were analyzed. The median plasma C_0_ values were 96.1 ng/mL (range 37.2–148.0 ng/mL) and 54.5 ng/mL (range 31.4–126.0 ng/mL), respectively (*P* = 0.066, Mann–Whitney U test) (Fig. 1b). Moreover, the bosutinib-induced liver dysfunction (≥ grade 1, or ≥ grade 2) was also observed in patients with high bosutinib C_0_ (> 91.0 ng/mL of quartile 4, calculated by the entire C_0_ distribution) (Table 4).

**Table 3:** Appearance of liver dysfunction and diarrhea within 180 days after treatment

Number of patients	Standard dose (n = 10)	Dose escalation (n = 15)	*P* value
Patient with liver dysfunction (> grade 1) (%)	5 (50.0%)	4 (26.7%)	0.234
Maximum grade (grade 2/3/4)	4/1/0	1/2/1	
Day of the appearance of liver dysfunction (> grade 1), median (range)	28 (19–57)	53.5 (35–112)	
Cumulative dose until the appearance of liver dysfunction over grade 2, mg, median (range)	8500 (6500–20,500)	11,300 (5500–36,300)	0.806
Dose at the appearance of liver dysfunction over grade 2, median (range)	400 (400–500)	350 (200–500)	0.241
Discontinuation of bosutinib due to liver dysfunction (%)	0 (0%)	1 (6.6%)	
Patient with diarrhea (all grade) (%)	10 (100%)	11 (73.3%)	0.075
Maximum grade (grade 0/1/2/3/4)	0/4/3/3/0	4/6/2/3/0	
Day of the first appearance of diarrhea, median (range)	1 (1–5)	19 (1–148)	< 0.0001
Dose at the first appearance of diarrhea (100/200/300/400/500 mg)	0/0/0/0/10	4/3/1/3/0	
Cumulative days of diarrhea, median (range)	20.5 (3–47)	6 (1–27)	0.104
Discontinuation of bosutinib due to diarrhea (%)	0 (0%)	0 (0%)

**Table 4:** Relationship between trough blood concentration and side effects observed in patients taking bosutinib

	Distribution of C_0_			*P*
	Q1	Q2–3	Q4	
Liver dysfunction				
≥ grade 1	6/34	29/68	15/34	0.019
≥ grade 2	0/34	2/68	4/34	0.050
≥ grade 3–4	0/34	1/68	2/34	0.231
Diarrhea				
≥ grade 1	5/34	8/68	6/34	0.714
≥ grade 2	1/34	2/68	5/34	0.041
≥ grade 3	1/34	0/68	4/34	0.012

*Q* quantile, *C*_*0*_ trough blood concentration. Q1 (≤ 32.5), Q2 (> 32.5), Q3 (≤ 91.0), Q4 (> 91.0). Median C_0_ = 54.0 (ng/mL)

Similarly, after administration of bosutinib, 10 of 10 patients (100%) experienced diarrhea between days 1 and 5 (median, day 1) on the SD regimen, as well as 11 of 15 (73.3%) between days 1 and 148 (median, day 19) on the DE regimen (Table 3). The Kaplan–Meier curve for the appearance of bosutinib-induced diarrhea is shown in Fig. 1c. The cumulative incidence of diarrhea was significantly higher in the SD group (*P* < 0.001, log-rank test). No patient discontinued bosutinib therapy due to diarrhea in either regimen. However, 11 out of 25 patients presented with delayed/prolonged diarrhea, which occurred after day 14. Significant differences of plasma C_0_ between patients with a delayed/prolonged diarrhea (on the day when max grade diarrhea occurred) and patients without diarrhea (on day 180) were identified. The median plasma C_0_ values were 103.5 ng/mL (range 58.3–144.5 ng/mL) and 55.5 ng/mL (range 31.4–126.0 ng/mL), respectively (*P* = 0.007, Mann–Whitney U test) (Fig. 1d). Moreover, diarrhea (≥ grade 2, or ≥ grade 3) was observed in patients with high bosutinib C_0_ (> 91.0 ng/mL of quantile 4, calculated by the entire C_0_ distribution) (Table 4). Although we tried to assess correlations between C_0_ of bosutinib and the clinical/laboratory data (age, sex, CML phase, reason of taking bosutinib, body weight, white blood cell count, hemoglobin levels, platelet count, aspartate transaminase, alanine transaminase, serum albumin levels, total bilirubin, serum creatinine levels, lipase value), there were no independent factors of the clinical characteristics influencing C_0_ (data not shown).

## Discussion

In the present study, the DE regimen, where dose was increased by 100 mg every 2 weeks, avoided treatment interruptions caused by adverse events, as compared to the SD regimen of the initial bosutinib dose of 500 mg/day. There were significant differences in the frequency of treatment interruption (90.0% vs. 13.3%, *P* < 0.001), as well as the duration of interruption of bosutinib (35 days vs. 14 days, *P * = 0.013), between the two regimens. In the SD regimen, 100% of patients had diarrhea within 5 days after beginning 500 mg/day of bosutinib and 50% of patients had liver dysfunction (> grade 1) by day 57 after beginning this regimen. This phenomenon was consistent with previous reports [1–3, 9–12]. However, diarrhea just after the initiation of bosutinib was not observed in patients on the DE regimen, except one patient (*P* < 0.0001), with 26.7% of patients on the DE regimen showing liver dysfunction (> grade 1) by day 112. There was a tendency of fewer patients showing liver dysfunction, or exhibiting a later symptomatic onset, on the DE regimen compared with the SD regimen, but this was not statistically significant (*P *= 0.234). As a result, using bosutinib therapy with a DE regimen, we could administer continuous treatment without interruptions, due to a reduction in the onset or duration of AEs.

The final dose of bosutinib during the 6-month treatment duration for both regimens was 343 and 346 mg/day, respectively, and the median cumulative dose during the treatment period was 51,700 and 53,550 mg, respectively. There were no significant dosimetric differences between the two treatment regimens. As a result of having adjusted the daily dose of bosutinib to minimize the incidence of adverse events, the median C_0_ during the treatment period for both regimens were the almost identical (63.7 and 63.0 ng/mL, respectively). In a phase I/II study investigating Japanese CML-CP patients, the mean daily dose was 339.3 mg (no data for C_0_) in a treatment period lasting 138 weeks [9], which is almost the same value as our present study.

This bosutinib C_0_ of 63 ng/mL in the present study was lower than the mean C_0_ of 156 ng/mL calculated by Hsyu et al. for an achievement of MMR at 12 months [8]. The mean C_0_ value reported by Hsyu et al. was obtained from a phase III trial of newly diagnosed CML-CP within 3 months after the administration of 500 mg/day of bosutinib [8]. Although the initial standard dose was 500 mg/day in this trial, dose reductions of bosutinib as a result of AEs occurred in 39% of the study patients [4], and the median daily dose was 489 mg/day [4, 8]. The exposure–response analysis for MMR and AEs have been reported for data from the BELA (n = 246) and BFORE (n = 266) trials [13]. The starting dose of bosutinib was 500 mg/day (BELA) and 400 mg/day (BFORE) [4, 5]. Although both trials had a higher proportion of Caucasian patients (79% vs. 64%) and a lower proportion of Asian patients (12% vs. 26%), the median C_0_ values of bosutinib were 67.51 ng/mL (C_0_ range 0.01–357.74) in the BELA trial and 55.75 ng/mL (C_0_ range 0.01–223.83) in the BFORE trial [13], which was similar to our results. The MMR rate at 12 months was 38.0% in the BELA trial and 47.2% in the BFORE trial for de novo CML patients, which was significantly higher than the control treatment using imatinib [4, 5]. Although we could not report any efficacy data in this study due to a short observation period, the importance of managing AEs in CML is underscored by the association between the AE occurrence and reduced treatment adherence. In addition, lower levels of adherence are associated with suboptimal treatment response [14]. The purpose of the dosage and treatment schedule investigated in our study was to reduce the occurrence of AEs and to avoid AE-related interruption in TKI treatment. A significantly higher number of patients were able to continue the bosutinib treatment in the DE regimen than the SD regimen. This result suggests that a higher level of adherence might be associated with an optimal treatment response.

The C_0_ value of 63 ng/mL may be appropriate in general clinical practice. The optimal therapeutic window for bosutinib is not yet determined; however, a target bosutinib C_0_ of 63 ng/mL in Japanese CML patients might be recommended to balance efficacy and toxicity. In addition, a bosutinib C_0_ of more than 91.0 ng/mL may be excessive for Japanese CML patients; the bosutinib-induced diarrhea > grade 2 and liver dysfunction > grade 1 were observed in patients with high bosutinib C_0_ (> 91.0 ng/mL).

Nakaseco et al. reported that bosutinib exposure on day 15 after administration is not different for daily doses of 500 and 600 mg [9], and Cortes et al. also reported that there is no increase in bosutinib exposure on day 15 when the bosutinib dose increased from 400 to 500–600 mg/day (mean AUC_0-24_: 2851; 3660; and 3360 ng*h/mL, respectively) [1]. These results show that the clinical response of bosutinib might depend on plasma concentration, but not the daily dose. In these reports, they explain that the solubility of bosutinib is saturated at a higher daily dose [1, 9]. In the previous study, the inter-individual variability of bosutinib was very large (% CV of about 70%) [8]. A possible causative factor for the large variability is a solubility of bosutinib due to a dependence on the intra-gastric pH, and it could continuously change the absorption of the drug from the gastrointestinal tract [15]. TDM for bosutinib might be a useful strategy for dose optimization to obtain a faster and deeper clinical response, due to the wide distribution of plasma concentrations for bosutinib.

The efficacy and safety of a dose-escalation schedule was confirmed by the bosutinib concentration level used in this study. The cost of the HPLC method for measuring the concentration of bosutinib is reasonable ($4 per sample, including running costs of HPLC) and it is easy to perform at any institute as demonstrated in the methods section [16]. However, the dosage of bosutinib can increase, according to the DE regimen, without measuring the concentration of bosutinib in daily practice. If patients show any AEs, even for low-grade AEs in the DE regimen, the dosage of bosutinib should be at the same level and treatment can be continued without interruption. If a clinician can monitor bosutinib C_0_, as provisionally defined by the ELN, simultaneously with an evaluation of clinical response according to transcript levels at each time point, then the mean bosutinib C_0_ should also be assessed for 3 months, or using multiple points, for each patient treated with bosutinib. In addition, after changing bosutinib dose, it may be necessary to monitor bosutinib C_0_ for approximately 1 week (day 8).

The limitation of this study was the small sample size. Further examination with a larger sample size will be necessary to evaluate the relationships between the pharmacokinetics of bosutinib and various AEs. However, individualization of the daily dose of bosutinib, based on C_0_, might be useful, as well as the TDM for other TKIs such as imatinib, nilotinib, dasatinib, and ponatinib [6, 17].

## Conclusions

The DE regimen was better than the SD regimen at avoiding treatment interruptions due to AEs. The daily dose of bosutinib might be adjusted based on the target C_0_ to avoid adverse events by TDM in general clinical practice.

## Abbreviations

TKI: tyrosine kinase inhibitor
Abl: Abelson
CP: chronic phase
CML: chronic myeloid leukemia
BELA: Bosutinib Efficacy and Safety in Newly Diagnosed CML
BFORE: First-line chrOnic myelogenous leukemia tREatment
MMR: major molecular response
FDA: Food and Drug Administration
TDM: therapeutic drug monitoring
AEs: adverse events
C_0_: trough plasma concentration
SD: standard dose
DE: dose escalation
CTCAE: Common Terminology Criteria for Adverse Events
HPLC: high-performance liquid chromatography
CVs: coefficient of variations
